# The use of behaviour management techniques amongst paediatric dentists working in the Arabian region: a cross-sectional survey study

**DOI:** 10.1007/s40368-020-00560-8

**Published:** 2020-09-09

**Authors:** H. Nazzal, O. I. El Shahawy, S. Al-Jundi, I. Hussein, J. F. Tahmassebi

**Affiliations:** 1 Paediatric Dentistry Section, Hamad Dental Centre, Hamad Medical Corporation, Doha, Qatar; 2 Department of Paediatric Dentistry, Faculty of Dentistry, Cairo University, Cairo, Egypt; 3 Faculty of Dentistry, Jordan University of Science and Technology, Irbid, Jordan; 4 Hamdan Bin Mohammed College of Dental Medicine, Mohammed Bin Rashid University of Medicine and Health Sciences, Dubai, United Arab Emirates; 5 Leeds School of Dentistry, Faculty of Medicine and Health, University of Leeds, Leeds, LS2 9LU UK

**Keywords:** Behaviour management, Children, Paediatric dentists, Arabian region

## Abstract

**Purpose:**

The purposes of this study were to investigate paediatric dental practitioners’ training and confidence in using dental behaviour management techniques in the Arabian region and to assess the factors influencing the application of advanced behaviour management techniques. Methods: An online questionnaire was distributed to paediatric dental practitioners in the Arabian region. Data were analysed using descriptive statistics and Pearson Chi Square.

**Results:**

A total of 113 responses were obtained. Of these, the majority were from Egypt (45%, *n* = 51). Just over half of the respondents were registered as specialists at the country where they were practicing paediatric dentistry (53%, *n* = 60). The use of behaviour management techniques varied amongst participants with tell-show-do (95%, *n* = 107) and positive reinforcement (89%, *n* = 101) being the most routinely used techniques. The majority of participants reported using voice control (83%) and parental separation (68%) techniques. Hand over mouth exercise (HOME) was only used by 24% (*n* = 27) of participants, whilst just over half of the participants, 53%, reported using protective stabilisation. A significant association was shown between country of practice, country of obtaining paediatric dental training, speciality status and the use of advanced behaviour management techniques, whilst confidence in using HOME and sedation were associated with work setting and country of practice, respectively.

**Conclusion:**

The use of advanced behaviour management techniques was found to be high amongst respondents in the Arabian region. The lack of training in using these techniques, however, is of concern. Further assessment of the factors affecting the use of and confidence in applying advanced behaviour management techniques in the Arabian region is needed.

## Introduction

The long-term success of any paediatric dental treatment is highly dependent on the child’s cooperation level. Therefore, paediatric dentists have to gain a good level of cooperation by applying various behaviour management techniques. The term behaviour management has been defined as ‘The means by which the dental health team effectively and efficiently performs treatment for a child’ (Wright 1975). Encouraging a positive lasting attitude to dentistry is as crucial as achieving the dental treatment. Managing the child’s behaviour in such a way that instils a positive dental attitude, not only would help improve the child’s future dental treatments, but would also aid to improve the child’s dental health.

Parental factors, such as child–parent relationship, parental anxiety, parent’s perception of children’s behaviour in the dental operatory, parent’s past dental experience and parents’ expectation of behaviour management, used by the dentist, have a major role in children’s behaviour during dental treatment (Suprabha and Rao 2015).

Different pharmacological and non-pharmacological behaviour management techniques exist with the purposes of either improving communication, eliminating inappropriate behaviour or reducing anxiety (Campbell et al. 2011). Non-pharmacological behaviour management employs a spectrum of techniques ranging from simple communication techniques, such as tell-show-do, to a more advanced aversive techniques, such as the use of the hand over mouth exercise (HOME). These techniques are usually used in combination either simultaneously or alternatively. The use of basic behaviour management techniques, such as tell-show-do, or positive reinforcement, is acceptable by the parents and seldom require explanation or consent. On the other hand, more advanced techniques, such as voice control or negative reinforcement, should be explained to parents in order to prevent parent’s misunderstanding and reduce future unnecessary litigations.

In recent decades, the focus on behavioural science in dentistry has been highlighted. Methodical and systematic undergraduate education in behavioural sciences, encompassing pharmacological and psychological methods, has been introduced in the dental schools in United Kingdom (UK) to help reduce and treat dental anxiety in both children and adults (Porritt et al. 2012, McDonnell-Boudra et al. 2014).

Few studies, in recent years, have explored why dentists from different countries may use one technique over another (Crossley and Joshi 2002; Adair et al. 2004; Campbell et al. 2011; American Academy of Pediatric Dentistry 2017). Parental perception and acceptance of different behaviour management techniques is one of the most important factors. Tell-show-do has been reported as the most accepted technique in most previous studies by dentists and parents (Crossley and Joshi 2002; Adair et al. 2004; Boka et al. 2014), whilst physical restraint and GA are usually reported as the least accepted techniques (Wright 1975; Adair et al. 2004; Boka et al. 2014). Culture plays an important role in parent’s decision and acceptance of different behaviour management techniques. Physical restraint/protective stabilisation, for instance, is used more frequently in USA., whilst general anaesthesia (GA) is more accepted in the UK (Crossley and Joshi 2002; Adair et al. 2004). In Saudi Arabia GA is a more acceptable approach by parents than physical restraint/protective stabilisation of uncooperative children requiring dental treatment (Abushal and Adenubi 2003).

There is dearth of literature reporting the use of different behaviour management techniques amongst paediatric dentists working in the Arabian region and the factors influencing such use. Therefore, the present study aimed at capturing paediatric dental practitioners’ use, training, experience and confidence in using paediatric dental behaviour management techniques in the Arabian region. The current study also aimed at assessing factors influencing paediatric dental practitioners’ use and confidence in applying advanced behaviour management techniques. This information would be valuable in improving our understanding of the type of behaviour management techniques used in the Arabian region and whether such techniques comply with current guidelines.

## Materials and methods

A 34-item online cross-sectional questionnaire was developed using the Online Survey tool (previously known as Bristol Online Survey). The survey was piloted, for ease of use and understanding, on a group of five specialist dentists. Ethical approval was obtained from the University of Leeds Research Ethics Committee before circulating the questionnaire (031218/JT/265). The questionnaire was circulated to Arabian Paediatric Dentists via the Arabian Academy of Paediatric Dentistry’s (ArAPD) Facebook page (https://www.facebook.com/ArAPD2015/), and the contact lists of the United Arab Emirates, Egyptian, Sudanese, Iraqi, Omani, and Libyan paediatric dental societies/clubs. In addition, the survey was also circulated through personal contacts to colleagues in Qatar, Bahrain, Saudi Arabia, Palestine and Jordan. This study was conducted between January and April 2019 with a reminder communication in March 2019. Due to the anonymity of the questionnaire, no individual follow-up was carried out.

The following information were collected:

- Demographic data, including, country of practice, gender, work setting, specialty status (general practitioner versus specialist) and country of specialty training;
- The type and nature of behaviour management training obtained;
- The frequency of using different basic behaviour management techniques, such as tell–show-do, modelling and positive reinforcement;
- The frequency, training, confidence and technique in obtaining parental consent when performing advanced behaviour management techniques, including parental separation, voice control, hand over mouth, protective stabilisation and use of Papoose Boards;
- Access, use and training in the use of sedation.

### Statistical analysis

Descriptive statistics were used in depicting demographic data and the frequency of using different behaviour management techniques. Pearson Chi Square test, with a significance level set at 0.05, was applied in assessing the association between the use of and confidence in applying advanced behaviour management techniques and respondents’ variables, such as country of practice, gender, work setting, specialty status and country of paediatric dental training. Due to the low number of participants representing certain variables, some variables were grouped together or eliminated.

## Results

A total of 115 responses were received. Of these, two participants were excluded (one working outside the Arabian region and one general practitioner not working in a paediatric dentistry post), resulting in 113 respondents and a response rate of 27.4%.

**Fig. 1:**
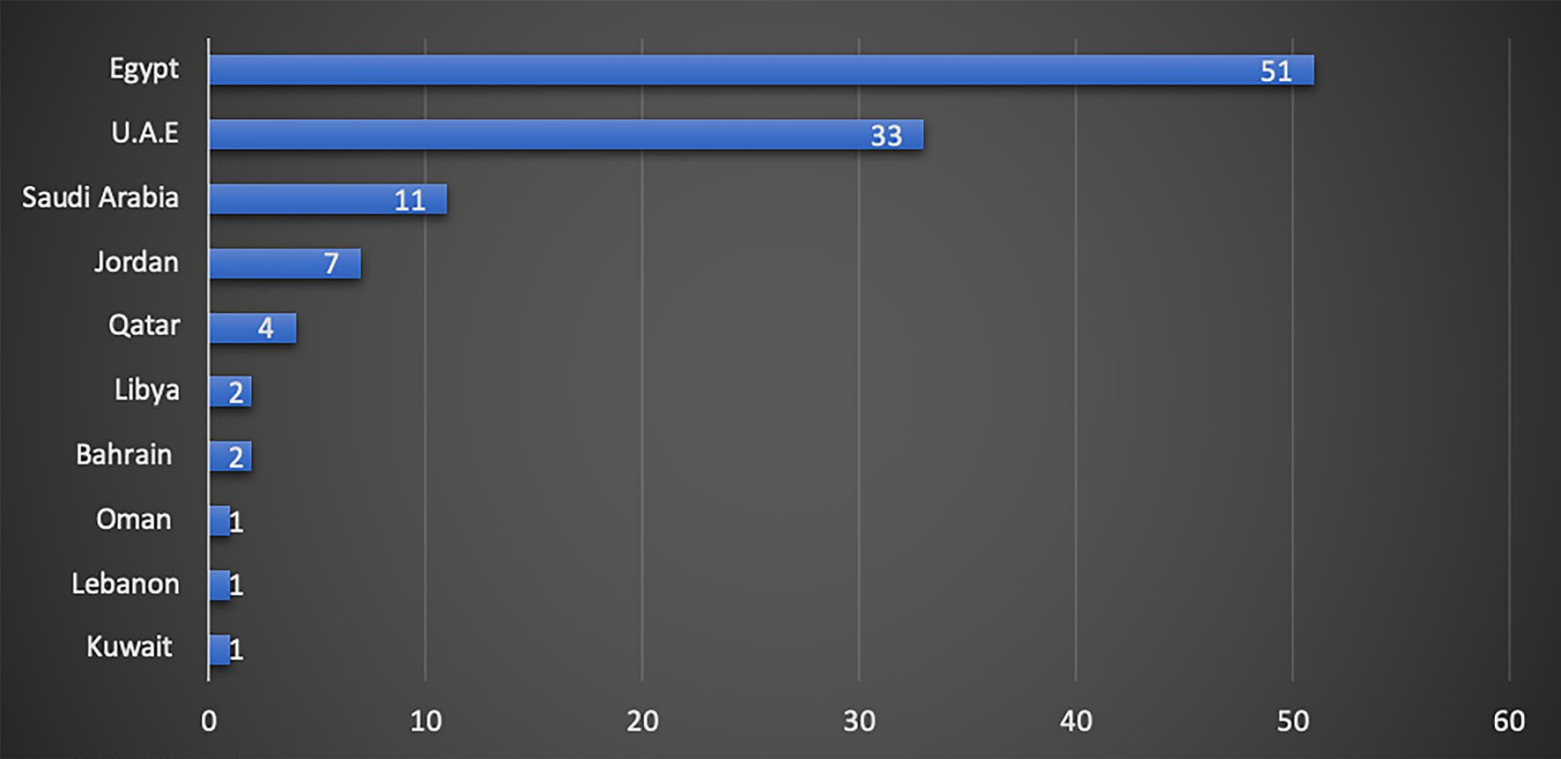
Bar chart showing participant’s country of practice

**Fig. 2:**
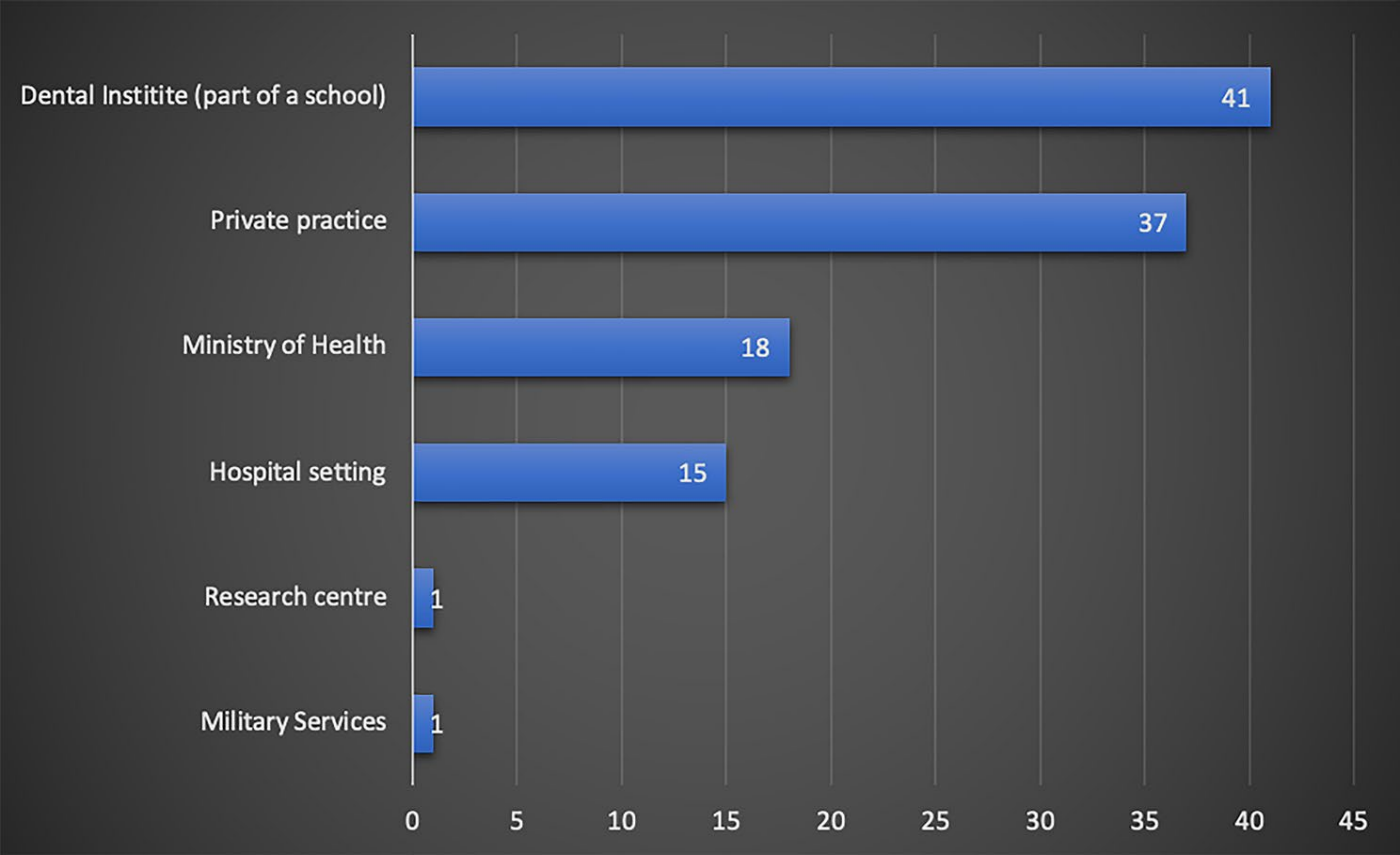
Bar chart showing type of participants’ place of work

**Fig. 3:**
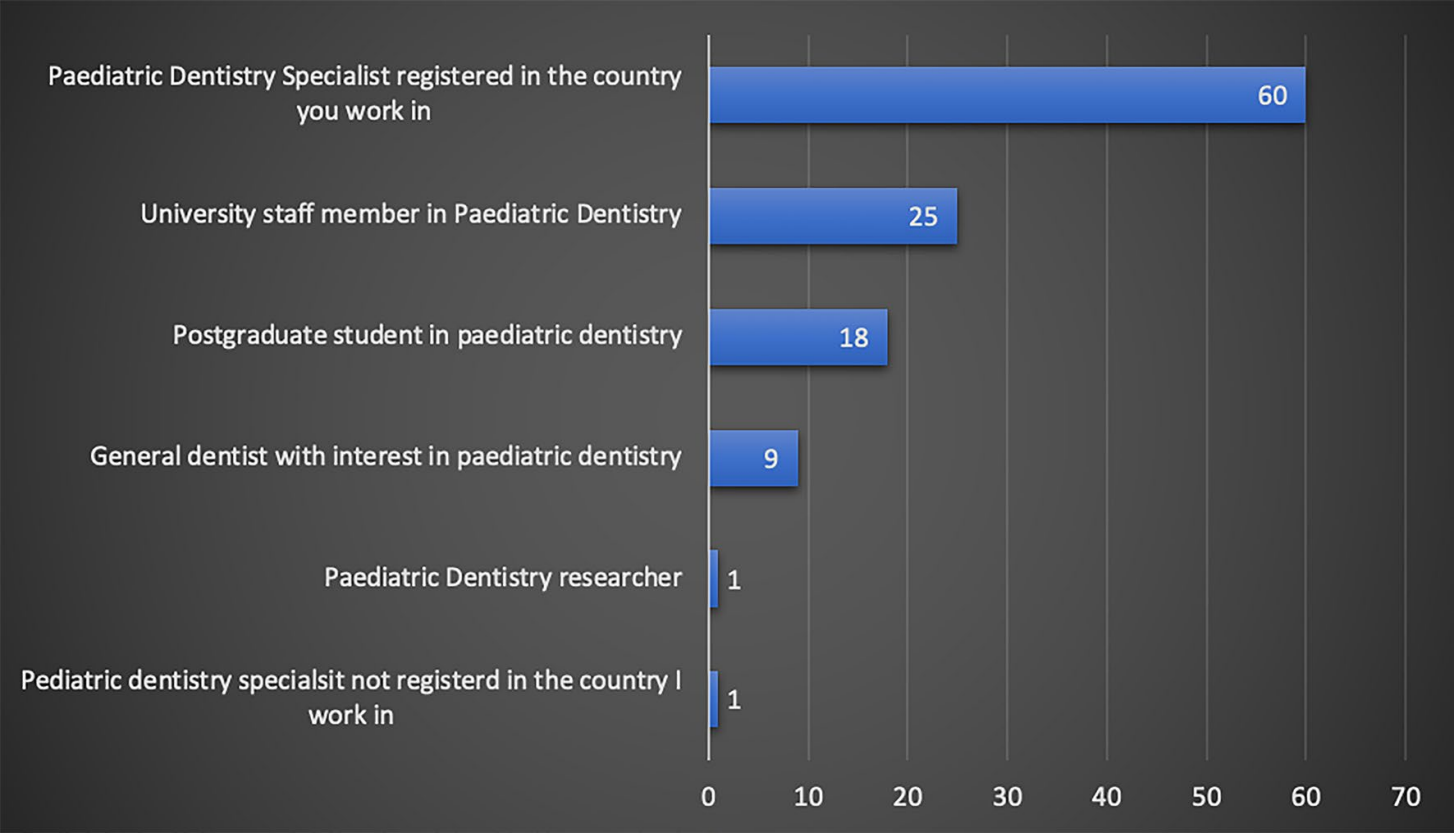
Bar chart showing participants’ paediatric dentistry speciality status

**Fig. 4:**
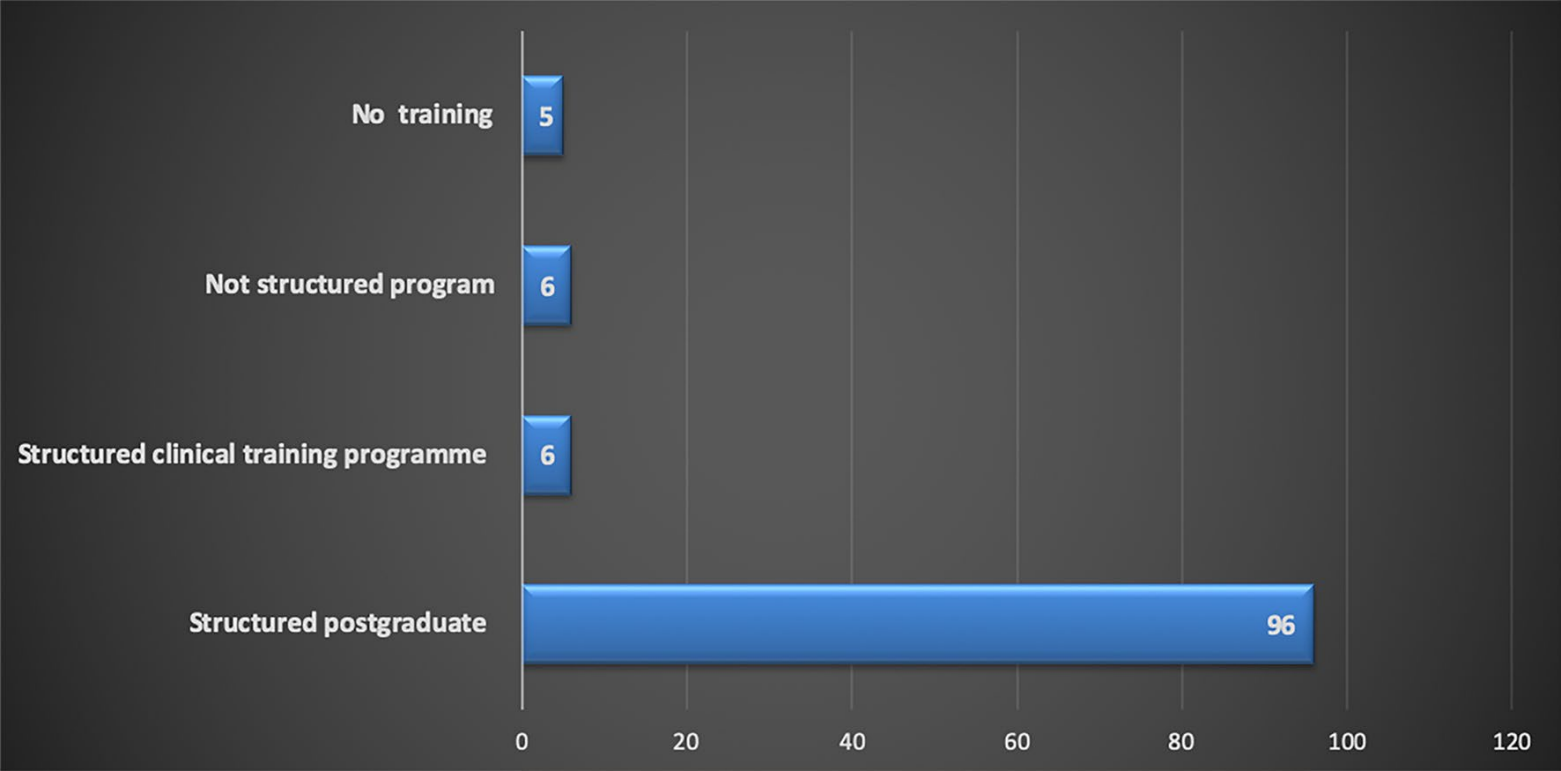
Bar chart showing the nature of respondent’s behaviour management training

Almost half of the respondents were from Egypt (45%, 51/113), with good representation from UAE (29%, 33/113), Saudi Arabia (9.7%, 11/113) and Jordan (6% 7/113) (Fig. 1). Almost a third of respondents worked at a dental institute/school (36%, 41/113), whilst the remaining respondents were mainly working in other settings, such as private practice, ministry of health and hospital settings (Fig. 2). Just over half of the respondents were registered as a specialist in paediatric dentistry at their country (53%, 60/113) (Fig. 3). The majority of participants obtained their paediatric training as part of a structured training programme (89%, 101/113). Interestingly, as shown in Fig. 4, a small proportion of respondents had no formal paediatric dental training (4%, 5/113).

**Fig. 5:**
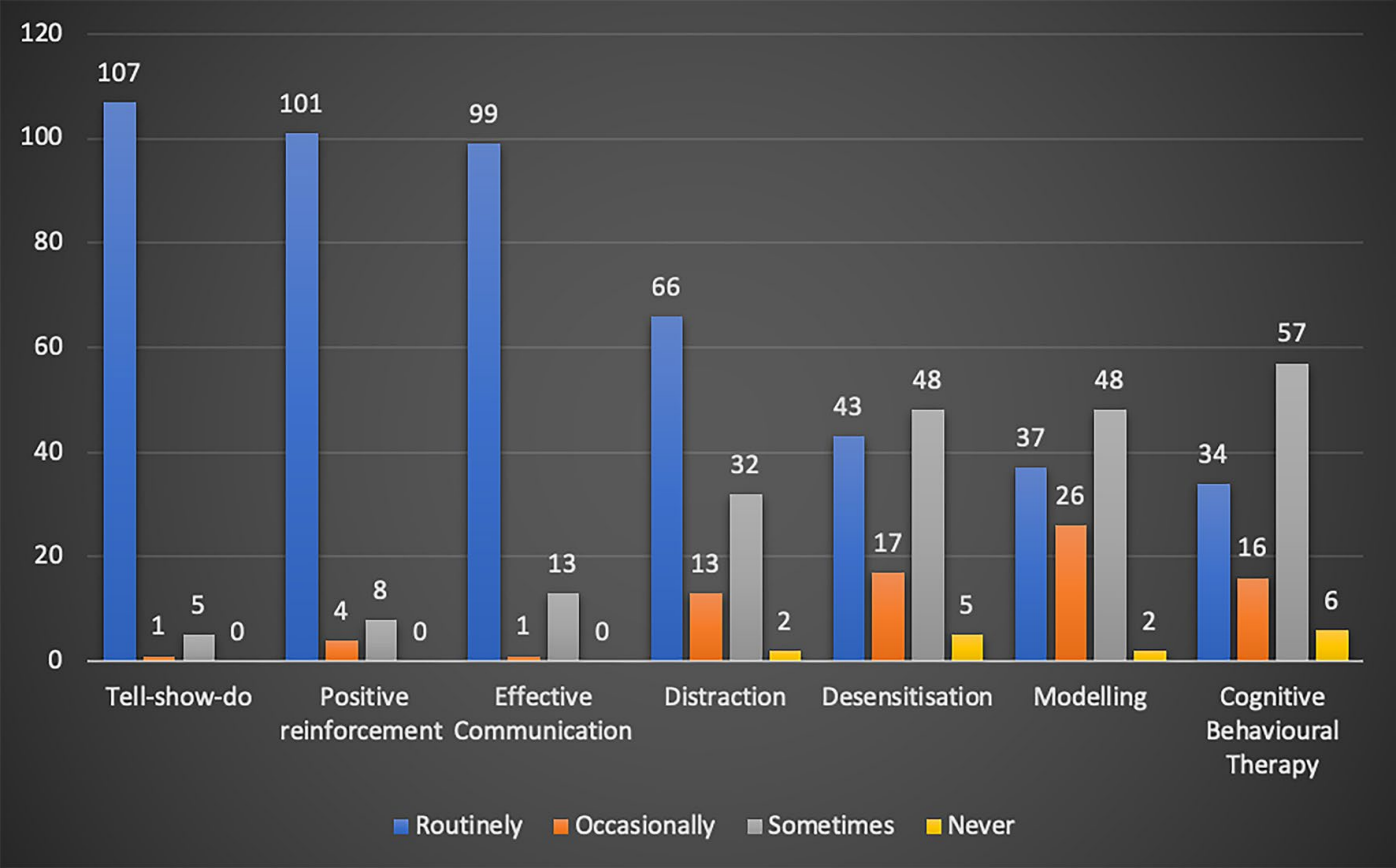
Bar chart showing use of basic behaviour management techniques amongst

The use of different behaviour management techniques varied amongst participants with tell-show-do, positive reinforcement and effective communication being the most routinely used techniques, whilst the use of more time demanding techniques, such as desensitisation, modelling and CBT was less frequent (Fig. 5).

The majority of participants reported using voice control (83%) and parental separation (68%) techniques. HOME, on the other hand, was only used by 24% (27/113) of participants, whilst just over half of the participants (53%) reported using protective stabilisation (Table 1).

**Table 1:** Participants’ use, level of training, clinician confidence and explanation/consent obtained before performing advanced behaviour management techniques

	Parental separation		Voice control		Home	
	*N*	%	*N*	%	*N*	%
Do you use this technique?						
No	36	31.9	19	16.8	86	76.1
Yes	77	68.1	94	83.2	27	23.9
Do you explain the technique and obtain consent before applying such technique?						
No	14	18.2	27	28.7	6	22.2
Yes-Routinely	37	48.1	38	40.4	12	44.4
Yes-Sometimes	23	29.9	25	26.6	7	25.9
Other	3	3.9	4	4.3	2	7.4
Have you had structured training in using this technique?						
No training	16	20.8	22	23.4	4	14.8
Yes-Not a structured programme	12	15.6	7	7.4	3	11.1
Yes-Structured programme	49	63.6	65	69.1	20	74.1
Do you feel confident using this technique?						
No	10	13.0	10	10.6	9	33.3
Yes	62	80.5	80	85.1	18	66.7
Sometimes	5	6.5	1	1.1	0	0.0
Other	0	0.0	3	3.2	0	0.0

Out of those who used HOME, 92% (25/27) were based in Egypt, 52% (14/27) worked at dental institutes and 89% (24/27) obtained paediatric dentistry training as part of structured programme. Interestingly three of those using HOME worked as university staff members in paediatric dentistry, however, with no training in paediatric dentistry over and above undergraduate training.

Protective stabilisation, on the contrary, was reported by participants based in different Arabian countries, such as Egypt (37%), UAE (28%), Saudi Arabia (17%), Jordan (7%), Qatar (5%) and 2% in Lebanon, Oman, Libya and Bahrain. This technique was used by participants working in different sectors with no apparent pattern. The majority of participants (88%, *n* = 53/60) who used this technique obtained structured paediatric dental training, whilst 7% (4/60) received non-structured training and 5% received no paediatric dental training.

**Fig. 6:**
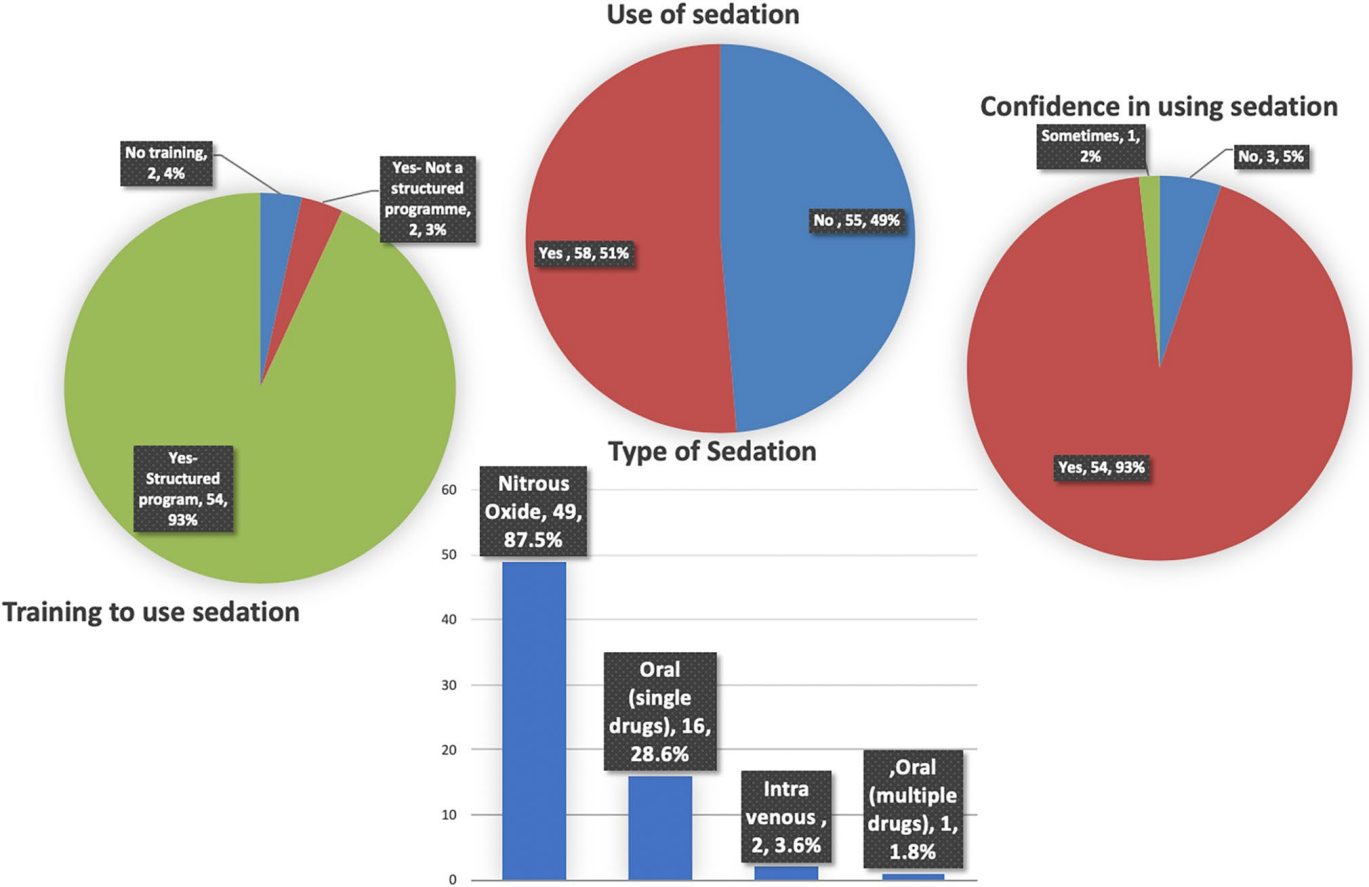
Charts showing the use of, type of, training obtained and confidence in using

The use of sedation, type of sedation, training obtained and confidence in using the technique is shown in Fig. 6. Almost half of the participants reported using sedation (51%, *n* = 58) with majority (87.5%, 49/58) using nitrous oxide inhalation sedation. Oral sedation was reported by 30.2% (17/58) of participants and intravenous sedation was used by only two participants. Four participants reported either no training or no structured training in using sedation with one of these participants reported using the oral sedation following self-training. These four participants reported lack of confidents using sedation.

Variables showing statistically significant association with the use of and confidence in applying advanced behaviour management techniques (parental separation, voice control, protective stabilisation and sedation) are presented in Table 2.

**Table 2:** Pearson’s Chi Square association between participant’s independent variables (country of practice, work setting, specialty status and country of speciality training) and the use of/confidence in using advanced behaviour management techniques (parental separation, voice control, protective stabilisation and sedation). Only Statistically significant associations are reported

			Use of					Confidence in	
			Parental separation	Voice control	HOME	Protective stabilisation	Sedation	HOME	Sedation
Country of practice	Pearson Chi Square	*P* value	0.026^£^	0.000^£^	0.000^£^	0.032^£^	0.000^£^		0.012^£^
		Value	8.709	23.079	28.139	8.644	49.769		9.939
	Saudi Arabia	Count	6	10	0	10	8		6
		% within Country	54.5%	90.9%	0.0%	90.9%	72.7%		75.0%
	Egypt	Count	41	50	25	22	11		10
		% within Country	80.4%	98.0%	49.0%	43.1%	21.6%		90.9%
	United Arab Emirates	Count	17	19	1	17	31		31
		% within Country	51.5%	57.6%	3.0%	51.5%	93.9%		100.0%
	Jordan	Count	5	6	1	4	2		1
		% within Country	71.4%	85.7%	14.3%	57.1%	28.6%		50.0%
Worksetting	Pearson Chi Square	*P* value	–	–	–	–	–	0.015^£^	–
		Value	–	–	–	–	–	8.491	–
	Hospital setting	Count	–	–	–	–	–	0	–
		% within working setting	–	–	–	–	–	0.0%	–
	Dental Institute/school	Count	–	–	–	–	–	12	–
		% within working setting	–	–	–	–	–	85.7%	–
	Private practice	Count	–	–	–	–	–	2	–
		% within working setting	–	–	–	–	–	28.6%	–
	Ministry of health	Count	–	–	–	–	–	4	–
		% within working setting	–	–	–	–		80.0%	–
Specialty status	Pearson Chi Square	*P* value	–	–	0.003^£^	0.024^£^	0.001^£^	–	–
		Value	–	–	13.406	9.262	15.789	–	–
	Registered Specialist	Count	–	–	9	29	31	–	–
		% within specialty status	–	–	15.5%	50.0%	53.4%	–	–
	University staff member	Count	–	–	13	19	11	–	–
		% within specialty status	–	–	50.0%	73.1%	42.3%	–	–
	Postgraduate student	Count	–	–	2	6	15	–	–
		% within specialty status	–	–	10.5%	31.6%	78.9%	–	–
	GDP with interest in paediatric dentistry	Count	–	–	3	6	0	–	–
		% within Specialty status	–	–	37.5%	75.0%	0.0%	–	–
Country of speciality training	Pearson Chi Square	*P* value	0.001^£^	0.000^£^	0.000^£^	–	0.000^£^	–	–
		Value	16.336	22.092	21.810	–	40.328	–	–
	Saudi Arabia	Count	4	4	0	–	4	–	–
		% within study country	100.0%	100.0%	0.0%	–	100.0%	–	–
	Egypt	Count	43	53	24	–	13	–	–
		% withinstudy country	79.6%	98.1%	44.4%	–	24.1%	–	–
	United Arab Emirates	Count	11	14	1	–	21	–	–
		% within study country	47.8%	60.9%	4.3%	–	91.3%	–	–
	Jordan	Count	7	8	0	–	3	–	–
		% within study country	87.5%	100.0%	0.0%	–	37.5%	–	–
	Non Arabian countries	Count	6	11	1	–	12	–	–
		% within study country	37.5%	68.8%	6.3%	–	75.0%	–	–

^£^Fisher’s Exact Test was used

The country of practice was the only variable significantly associated with all advanced behaviour management techniques (parental separation, voice control, protective stabilisation, and sedation) (*p* < 0.05) (Table 2). Except for the use of sedation and protective stabilisation, respondents working in Egypt were more likely to use advanced behaviour management techniques than those working in other Arabian countries (Table 2).

The country of speciality training was significantly associated with the use of all advanced techniques except for protective stabilisation (*P* < 0.05) (Table 2). Respondents trained in Saudi Arabia were 100% likely to use protective stabilisation, voice control and sedation, whilst none reported using HOME in managing children’s behaviour (Table 2). Respondent’s speciality status was found to be significantly associated with the use of HOME, protective stabilisation and sedation (Table 2). Lack of confidence in using sedation was least reported in those practising sedation in Egypt (9.1%).

## Discussion

This study was the first to survey the use of different behavioural management techniques in paediatric dentistry in a large part of the Arabian region. The lack of formal published data accurately reporting the total number of paediatric dental specialists in the Arabian region prevented the calculation of an exact response rate. Therefore, the number of Arabian paediatric dentists was estimated based on the method described by Hussein et al. (2020) in which approximately 68000 paediatric dentists are serving 7.7 billion people world-wide; therefore, using proportional calculation, around 3700 paediatric dentists would serve the Arabian population of 423 million. This estimate is very close to the number of the ArAPD Facebook page members of 3726 whom the survey was disseminated to. Consequently, a sample size calculation with a 95% confidence level of 95% and 5% margin of error yielded a sample size of 349. Adding to that a 20% of no responses (70), the final sample size calculation reached was 419. Based on this estimate, a response rate of 27.4% of the estimated sample size was achieved by this survey study. This response rate is in line with other published dental and medical surveys showing a response rate between 20 and 32% (Cunningham et al. 2015; Chyou et al. 2017). Nevertheless, this study attracted participants from almost half of the countries in the Arabian region (10/21 countries, 45%). The majority of respondents were from Egypt, UAE and Saudi Arabia. These three countries have the largest populations in the Arabian region and hence have a greater number of available paediatric dentists or paediatric dental practitioners than the other less populated countries.

There was a good representation of practitioners from different clinical sectors in the current study with more participants working in dental institutes. In addition, a range of paediatric dental practitioners completed this survey, with almost half of the participants at a level of paediatric dental specialists registered in the country of practice. Participants demonstrating further training in paediatric dentistry and/or working in a paediatric dentistry post were included in this study. Interestingly, five participants, working as university staff members, reported having no paediatric dental training over and above that obtained at undergraduate level.

Tell-show-do and positive reinforcement are two of the most successful yet simple basic behaviour management techniques which can be used with all paediatric patients regardless of their cooperation level (American Academy of Pediatric Dentistry 2017). In the present study, these two techniques were found to be the most popular techniques. A recent survey of members of the American Academy of Paediatric Dentistry (AAPD) reported similar popularity (99%) with both techniques. Tell-show-do-based techniques, such as tell-play-do and ask-tell-ask, have recently been introduced and recommended as behaviour management techniques (American Academy of Pediatric Dentistry 2017; Vishwakarma et al. 2017). These techniques were not included in the present survey in order to reduce the number of survey questions.

Distraction is also a simple and effective behaviour management technique that could be used with any child regardless of their cooperation level (American Academy of Pediatric Dentistry 2017). Although the routine use of such technique is less than tell-show-do and positive reinforcement, the results of the current study are in line with other reported surveys whereby distraction has been reported to be used by the majority of respondents (Adair et al. 2004).

A high level of dental anxiety of around 22% of children in the Arabian region has been reported (Alshoraim et al. 2018; AlGharebi et al. 2020). Desensitising, modelling and CBT techniques are thus useful techniques in the management of anxious children and those with specific dental phobias (Stokes and Kennedy 1980; Campbell et al. 2011; Gomes et al. 2018). The specific indications, preparation and time consumption required for such techniques are likely reasons for the lower frequency of use reported in the current study. Desensitisation and cognitive behavioural therapy usually involves multiple patient contact in order to systematically help the children overcome their fear or phobias (Campbell et al. 2011), whilst modelling require preparation of a suitable patient model or modelling material (Melamed et al. 1975).

Although voice control is classified as a basic behaviour management technique, appropriate training and application is crucial for the success of such technique and avoidance of unnecessary patient and parent’s distress. The use of voice control in the Arabian region was found to be similar to that reported by members of the AAPD (92%) (Adair et al. 2004).

Even though the use of HOME is no longer recommended by the British Society of Paediatric Dentistry (BSPD) (Campbell et al. 2011) and the AAPD (American Academy of Pediatric Dentistry 2017), this technique was reported to be used by almost a quarter of respondents in the current survey. Such difference might be associated with cultural differences or difficulties in accessing GA services in some Arabian countries in comparison to UK and USA. Provision of paediatric dental treatment under GA is generally freely available for medically compromised and fit and healthy children in all participating countries except Kuwait (not available for fit and healthy children) and Egypt (provided by some universities and funded by charity organisations).

Protective stabilisation is a technique that is recommended in specific situations, such as when immediate diagnosis and/or urgent limited treatment is needed in uncooperative patients, and when patients pose a risk of harm to staff/parents especially where the use of sedation/GA is not possible (American Academy of Pediatric Dentistry 2019–2020). The current survey shows a wide use of protective stabilisation in the Arabian region with much lower proportion reporting the use of Papoose Boards as a form of passive restraint. These results are within the range reported by members of the AAPD in which passive stabilisation and active stabilisation were reportedly used by 68% and 73% in non-sedated patients and 56% and 47% in sedated patients, respectively (Adair et al. 2004). The use of active and passive restraint has been reported as the least popular technique amongst UK paediatric dentists with 69% and 61% reported being uncomfortable using active restraint and Papoose Boards, respectively (Crossley and Joshi 2002). Such results might again be linked to the availability of GA option in the UK in comparison to the Arabian region and the exclusion of GA from most dental insurance schemes in USA.

Interestingly, despite the agreement amongst most guidelines that stabilisation should be used in urgent short procedures (American Academy of Pediatric Dentistry 2019–2020), seven participants, in the present study, reported using Papoose Boards whilst providing any dental treatment regardless of time requirements. This might be the result of lack of appropriate training reported by 37% of respondents in this study. The use of restraint/protective stabilisation should only be used by properly trained personnel after obtaining informed consent for the provision of urgent short treatments.

Lack of training, confidence, appropriate explanation and consent prior to the use of voice control, parental separation, HOME and protective stabilisation have been highlighted in this study. To the authors’ knowledge this is the only study assessing training and confidence amongst paediatric dentists in using such advanced techniques. The confidence in using advanced behaviour management techniques was not associated with most of the respondent’s variables. The association reported between sedation per country of practice and HOME per work setting showed no specific pattern. The level of comfort whilst using different behaviour management techniques, rather than confidences, has been reported amongst UK paediatric dentists. According to this survey, UK paediatric dentists were mostly uncomfortable using Papoose Boards (98%), HOME (97%), active restraint (96%) and voice control (31%) (Crossley and Joshi 2002).

The BSPD and AAPD guidelines recommend that practitioners, using such advanced techniques, should obtain structured training, such as that obtained through residency programmes, graduate programmes and/or an extensive continuing education courses. Self-training, as mentioned by one of the respondents, might be acceptable with basic techniques, such as tell-show-do, but should not be acceptable with advanced techniques, such as protective stabilisation.

Obtaining informed consent, prior to the use of advanced management techniques, has been recommended with written consent recommended for protective stabilisation (Campbell et al. 2011; American Academy of Pediatric Dentistry 2019–2020). Pre-treatment explanation of such techniques would help secure parental cooperation during the session and reduce post-treatment complaints.

Sedation, in the form of nitrous oxide sedation, was found to be widely used in the Arabian region. Better compliance with training, confidence and consent was evident with the use of sedation in comparison to other non-pharmacological management techniques. Alarmingly, very small proportion of the respondents reported using such technique with lack of confidence and training. Health authorities in some Arabian countries prohibit the use of sedation in dentistry, such as the State of Qatar, which affected the overall use of sedation in the Arabian region.

The country of practice and country of obtaining paediatric dental education were associated with the majority of advanced behaviour management techniques. This is likely influenced by the differences in local culture, population size, parental acceptance, cost and availability of pharmacological behaviour management techniques, such as sedation and GA. The greater use of advanced behaviour management techniques in Egypt, for instance, could be related to the country’s population size, availability of sedation/GA and financial implications in low socioeconomic areas. The reported lack of HOME training by participants trained in Saudi Arabian and Jordan is likely the cause for the lack/lower number of reported use of such technique in the same countries. Further assessment of the factors affecting the use of and confidence in using advanced behaviour management techniques in the Arabian region.

## Conclusions

The results of the present study highlighted the use of a variety of behavioural management techniques amongst paediatric dental specialists and dentists with interest in paediatric dentistry working in the Arabian region. The use of advanced behavioural management techniques, such as parental separation, HOME and protective stabilisation, are relatively high amongst respondents. The lack of training and confidence in using such advanced behavioural management techniques, amongst a proportion of respondents, is of concern. Paediatric dentists and dentists working in the capacity of paediatric dentists should obtain structured training in the use of more advanced behaviour management techniques before employing such techniques. Paediatric dental societies/clubs in the Arabian region should work closely with local authorities in order to streamline the use of these techniques through the production of local guidelines and laws prohibiting the use of such techniques without appropriate training.

## Data Availability

Not applicable.
