# Correction to: The use of behaviour management techniques amongst paediatric dentists working in the Arabian region: a cross-sectional survey study

**DOI:** 10.1007/s40368-022-00713-x

**Published:** 2022-05-23

**Authors:** H. Nazzal, O. I. El Shahawy, S. Al-Jundi, I. Hussein, J. F. Tahmassebi

**Affiliations:** 1 Paediatric Dentistry Section, Hamad Dental Centre, Hamad Medical Corporation, Doha, Qatar; 2 Department of Paediatric Dentistry, Faculty of Dentistry, Cairo University, Cairo, Egypt; 3 Faculty of Dentistry, Jordan University of Science and Technology, Irbid, Jordan; 4 Hamdan Bin Mohammed College of Dental Medicine, Mohammed Bin Rashid University of Medicine and Health Sciences, Dubai, United Arab Emirates; 5 Leeds School of Dentistry, Faculty of Medicine and Health, University of Leeds, Leeds, LS2 9LU UK

## Correction to: European Archives of Paediatric Dentistry (2021) 22:375–385 10.1007/s40368-020-00560-8

After publication in Vol 22 Issue 3, it was noticed that the affiliation of author S. Al-Jundi was incorrectly given as University of Science and Technology. The correct affiliations are given in the contact details section.

3. Faculty of Dentistry, Jordan University of Science and Technology, Jordan.

